# Distinct epigenetic and gene expression changes in rat hippocampal neurons after Morris water maze training

**DOI:** 10.3389/fnbeh.2015.00156

**Published:** 2015-06-16

**Authors:** Sylvia D. Carter, Karen R. Mifsud, Johannes M. H. M. Reul

**Affiliations:** Neuro-Epigenetics Research Group, School of Clinical Sciences, University of BristolBristol, UK

**Keywords:** hippocampus, Morris water maze, histone H3 phosphorylation, immediate early genes, memory, chromatin immuno-precipitation, cognition, stress

## Abstract

Gene transcription and translation in the hippocampus is of critical importance in hippocampus-dependent memory formation, including during Morris water maze (MWM) learning. Previous work using gene deletion models has shown that the immediate-early genes (IEGs) c-Fos, Egr-1, and Arc are crucial for such learning. Recently, we reported that induction of IEGs in sparse dentate gyrus neurons requires ERK MAPK signaling and downstream formation of a distinct epigenetic histone mark (i.e., phospho-acetylated histone H3). Until now, this signaling, epigenetic and gene transcriptional pathway has not been comprehensively studied in the MWM model. Therefore, we conducted a detailed study of the phosphorylation of ERK1/2 and serine10 in histone H3 (H3S10p) and induction of IEGs in the hippocampus of MWM trained rats and matched controls. MWM training evoked consecutive waves of ERK1/2 phosphorylation and H3S10 phosphorylation, as well as c-Fos, Egr-1, and Arc induction in sparse hippocampal neurons. The observed effects were most pronounced in the dentate gyrus. A positive correlation was found between the average latency to find the platform and the number of H3S10p-positive dentate gyrus neurons. Furthermore, chromatin immuno-precipitation (ChIP) revealed a significantly increased association of phospho-acetylated histone H3 (H3K9ac-S10p) with the gene promoters of c-Fos and Egr-1, but not Arc, after MWM exposure compared with controls. Surprisingly, however, we found very little difference between IEG responses (regarding both protein and mRNA) in MWM-trained rats compared with matched swim controls. We conclude that exposure to the water maze evokes ERK MAPK activation, distinct epigenetic changes and IEG induction predominantly in sparse dentate gyrus neurons. It appears, however, that a specific role for IEGs in the learning aspect of MWM training may become apparent in downstream AP-1- and Egr-1-regulated (second wave) genes and Arc-dependent effector mechanisms.

## Introduction

Hippocampus-dependent long-term memory formation is important for allowing adaptation and optimization of behavioral responses when similar circumstances are encountered again. The Morris water maze (MWM) paradigm is frequently used as a model to study spatial memory formation, which involves *de novo* gene transcription and protein synthesis in the hippocampus. Glucocorticoids secreted in response to a stressful event, such as being placed into the MWM, facilitate hippocampal learning and memory processes (Oitzl and De Kloet, 1992; Sandi et al., 1997; Oitzl et al., 2001; Shors, 2001). Research into the molecular changes underlying hippocampus-dependent memory formation has identified an important role of the ERK MAPK (extracellular signal-regulated kinase, mitogen-activated protein kinase) cascade and subsequent induction of the immediate-early genes (IEGs) FBJ murine osteosarcoma (c-Fos), early growth response 1 (Egr-1) and activity-regulated cytoskeleton-associated protein (Arc) (Atkins et al., 1998; Blum et al., 1999; Guzowski et al., 2001; Adams and Sweatt, 2002). Studies using Arc, c-Fos, and Egr-1 knock-out mice, as well as antisense oligodeoxynucleotides, have indicated a necessity for these IEGs in the consolidation of hippocampus-dependent memory (Paylor et al., 1994; Guzowski et al., 2000; Jones et al., 2001; Guzowski, 2002; Plath et al., 2006; Czerniawski et al., 2011).

IEG induction is thought to be regulated by ERK MAPK signaling, however, the exact underlying molecular mechanisms are presently unknown. Activation of the ERK MAPK cascade results in recruitment of nuclear kinases and transcription factors such as Elk-1 and CREB (Xia et al., 1996; Deak et al., 1998; Davis et al., 2000) as well as chromatin modifying enzymes [mitogen- and stress-activated kinase (MSK) and histone acetyl transferases (HATs)], leading to epigenetic changes within the chromatin in sparse dentate gyrus granule neurons (Chwang et al., 2007; Chandramohan et al., 2008; Reul et al., 2009, 2015; Gutierrez-Mecinas et al., 2011; Reul, 2014). The resultant phosphorylation of serine-10 and acetylation of lysine residues within histone H3 is thought to de-condense (open) the chromatin structure, potentially allowing transcription factors to bind to their responsive elements, leading to transcription of formerly silenced genes (Nowak and Corces, 2000; Cheung et al., 2000a,b; Reul et al., 2009; Mifsud et al., 2011; Trollope et al., 2012).

The functional significance of these molecular changes in dentate gyrus neurons during stress-related memory formation has been studied using the forced swim paradigm. In this test, rats and mice show a passive, adaptive response of increased behavioral immobility when re-exposed to forced swimming. Pre-treatment of rats with either an N-methyl D-aspartate receptor (NMDAR) antagonist, a glucocorticoid receptor (GR) antagonist or an ERK MAPK kinase (MEK) inhibitor, or using MSK1/2 double knockout mice, each blocked histone H3 phospho-acetylation and IEG induction in dentate granule neurons and the consolidation of the behavioral immobility response in a 24 h (or even 4-week) forced swim re-test (De Kloet et al., 1988; Chandramohan et al., 2008; Gutierrez-Mecinas et al., 2011).

These ERK MAPK-driven epigenetic modifications may also play a role in IEG induction during memory formation of more pro-active behavioral responses, such as those during spatial MWM learning. However, at present the phosphorylation of ERK1/2, formation of the combinatorial, phosphorylated and acetylated, histone H3 marks, and induction of the IEGs c-Fos, Egr-1, and Arc in neurons of the different sub-regions of the dorsal hippocampus has not been investigated within the context of MWM learning. Moreover, the existence of a direct association of this double histone mark with IEG promoters following MWM training has not been studied either. This study, therefore, provides a detailed hippocampal sub-region specific time course of ERK1/2, and H3S10 phosphorylation and c-Fos, Egr-1, and Arc induction in response to MWM training. Changes in these factors in MWM trained rats have been compared with time-matched swim controls as well as naïve baseline animals. Finally, using chromatin immuno-precipitation (ChIP) we determined the association of the combinatorial histone H3 phospho-acetylation mark with the gene promoters of the IEGs c-Fos, Egr-1 and Arc in response to training in the MWM.

## Materials and methods

### Animals

Male Lister Hooded rats (140–175 g on arrival) were purchased from Harlan (Oxon, UK) and group-housed (2–4 per cage). Rats were housed under standard lighting (lights on 05:00–19:00 h, approximately 100 Lux) and environmentally controlled conditions (temperature 21 ± 1°C; relative humidity 40–60%) with food and water available *ad libitum*. All procedures were approved by the University of Bristol Ethical Committee and by the Home Office of the UK. All rats were handled for at least 5 days prior to experimentation in order to reduce non-specific handling stress on the day of the experiment.

### Behavioral procedures

The MWM set-up consisted of a circular, stainless steel tank (1.8 m diameter) filled with water (25°C ± 1) to a depth of approximately 30 cm. The maze was divided into four virtual quadrants and a transparent platform was placed in the center of the north-east quadrant, submerged 2 cm below the surface of the water. Visual cues were placed on the walls surrounding the maze. Rats were trained to find the platform, which was kept in a fixed position throughout training. Rats received one training session per day, each comprising four training trials. On the first day only, training trials were preceded by an acclimatization trial, whereby rats were allowed to explore the maze for 3 min with no platform present. Each training trial was a maximum of 3 min long, with an inter-trial time of at least 10 min, during which rats were returned to their home cage. For each of the four training trials the rat was placed into the maze, facing the edge, from a different direction (north, east, south, and west). A trial was deemed to be completed if the rat had found the platform, climbed on to it and remained there for 20 s. If the rat had not found the platform within the 3 min, it was guided to the platform by hand. Fully trained rats received one training session per day for 4 days, followed by a probe trial on the fifth day. For the probe trial, rats were placed into the maze for 3 min in the absence of the platform, during which the number of platform zone crossings and the latency to the first platform zone crossing were recorded. After each training session, rats were returned to their home cages, with the same housing conditions as prior to training. For mRNA, protein and ChIP analyses, groups of rats received training on only 1 day and were killed at varying points during and following training. Behavior throughout training was recorded using the Noldus EthoVision video tracking system, version 3 (Noldus Information Technology, Wageningen, Netherlands).

Two control groups were used in our study: (1) Baseline (BL), i.e., naïve rats, killed directly from their home cages; (2) Swim control (SC) rats that swam in the same maze as MWM trained rats and were matched to the MWM condition for both the number and the length of trials, but with no platform present in the maze during their trials. Swim control rats were therefore free to swim and explore the maze for equal amounts of time but there was no active platform location learning for this group.

### Immunohistochemistry

For immunohistochemistry, rats were deeply anesthetized with isoflurane, given a lethal injection of pentobarbital and transcardially perfused with 100 ml ice-cold 0.9% saline followed by 500 ml of 4% (w/v) paraformaldehyde containing 1 mM sodium orthovanadate (Na_3_VO_4_) and 50 mM sodium fluoride (NaF) and 0.195% picric acid in 0.1 M phosphate buffer (PB). Perfused brains were then extracted from the skull and stored at 4°C in 0.1 M phosphate buffer (PB) containing 1 mM Na_3_VO_4_ and 50 mM NaF. Unless otherwise stated, all reagents for this study were purchased from Sigma-Aldrich (Dorset, UK).

Separate groups of rats were killed under baseline conditions, immediately after each training trial, as well as at 2, 4, and 8 h after the start of MWM training. Perfused rat brains were cut into 50 μm thick coronal sections using a vibratome (Leica VT 1000S) and the avidin-biotin complex (ABC) method was used to visualize pERK1/2, H3S10p, c-Fos, Egr-1, and Arc in free-floating sections spanning the dorsal hippocampal region [between coordinates (anterior-Posterior) −2.92 and −3.96 mm from Bregma, according to the atlas of Paxinos and Watson, 2007]. For H3S10p and pERK1/2 staining an initial antigen retrieval step was carried out and all solutions up to and including the primary antibody solution contained 50 mM NaF and 1 mM Na_3_VO_4_ to inhibit endogenous phosphatases. For H3S10p staining, sections were initially incubated with 1N HCl at 45°C for 30 min. For pERK1/2, sections were initially incubated with citrate buffer at room temperature (RT) for 90 min. In both cases, sections were then washed with 0.1 M PB with phosphatase inhibitors. All other steps were carried out at RT.

For all antibodies, sections were incubated for 1 h in blocking solution (50 mM glycine, 2% Bovine Serum Albumin (BSA), 0.05% sodium azide and 0.1% Triton X-100 in 0.1 M PB) followed by an overnight incubation with primary antibody; either rabbit polyclonal anti-pERK1/2 [1:200, New England Biolabs (NEB)], rabbit monoclonal anti-phospho histone H3 (S10; 1:400, NEB), rabbit polyclonal anti-c-Fos (1:10,000, Calbiochem, Merck), rabbit monoclonal anti-Egr-1 (1:1000, NEB) or rabbit anti-Arc (1:1000, kindly donated by Dr. P. Worley, John Hopkins University, Baltimore) in incubation solution (0.8% BSA, 0.1% Triton X-100 and 0.05% sodium azide in 0.1 M PB). The anti-H3S10p has been used by us before and generates a highly similar staining pattern of sparse dentate gyrus neurons as the anti-H3S10p-K14ac antibody (Chandramohan et al., 2007, 2008) and the anti-H3K9ac-S10p antibody (Mifsud and Reul, unpublished observations). Unfortunately, the H3S10p-K14ac antibody, still commercially available, has lost its specificity for this combinatorial mark in recent years and is not used by us anymore. Sections were then washed in 0.1 M PB and incubated in biotinylated goat anti-rabbit IgG secondary antibody (1:400 in 0.1 M PB; Vector laboratories) for 1–2 h. This was followed by further washes with 0.1 M PB and then incubation with avidin-biotinylated peroxidase complex (ABC, 1:400 in 0.1 M PB, Vector Laboratories) for 1 h. The sections were washed again with 0.1 M PB and the reaction was developed using a 0.03% solution of 3,3′-diaminobenzidine tetrahydrochloride (DAB) with 0.003% nickel and 0.04% ammonium chloride in 0.1 M PB and catalyzed with 0.003% hydrogen peroxide. Following development, sections were mounted onto poly-L-lysine coated slides and dehydrated with ethanol. Slides were then cover slipped using Histomount (National Diagnostics).

### RNA analysis

For RNA analysis, the rats were quickly anaesthetised with isoflurane (Merial Animal Health Ltd.) and decapitated; brains were then removed, cut into 1 mm coronal slices using a brain matrix after which these slices were arranged on a stainless steel box filled with ice. The dentate gyrus (DG) and cornu ammonis (CA) regions of the dorsal hippocampal area were separately dissected from surrounding tissue, using Dumont #7 forceps (Fine Science Tools, Vancouver, Canada), under a dissection microscope (Leica M29_5_, Leica biosystems), as previously published (Kersante et al., 2013). The region microdissected spanned coordinates (anterior-Posterior) −2.92 and −3.96 mm from Bregma, according to the atlas of Paxinos and Watson (2007). Microdissected DG and CA tissue was snap-frozen in tubes in dry ice and stored at −80°C.

Rats were killed at baseline, or 30 min, 1, 3, or 7 h after the start of MWM training or SC experiences. Dorsal DG and CA region tissue samples were homogenized and RNA was extracted using TRI reagent (1 ml for CA regions, 500 μl for DG) as per manufacturer's instructions. The resulting RNA pellets were dissolved in 30 μl of nuclease free water (Ambion). RNA integrity was confirmed by running samples on a 1% agarose gel with 0.005% ethidium bromide. Selected samples were also analyzed for integrity with an Agilent 2100 Bioanalyser (Agilent Technologies); all RNA samples analyzed in this way had RNA integrity numbers of between 8.40 and 9.60. RNA concentrations were determined using a NanoPhotometer Pearl (Implen). Total RNA was converted to cDNA using a QuantiTect reverse transcription kit (Qiagen, Manchester, UK) as per the manufacturer's instructions, which included a step to remove DNA contamination.

Quantitative PCR (qPCR) was used to quantify the mRNA levels of target genes, using primers and dual-labeled probes modified with FAM as the fluorescent dye and TAMRA as the quencher. A combination of *ActB*, *Yhwaz*, and *Hprt1* were used as reference genes. Primers and probes were designed using primer express software (version 3.0, Life Technologies). See Table 1 for cDNA primer and probe sequences. In a 96-well plate, 2 μl of cDNA was added to 18 μl of reaction solution [1x TaqMan fast advanced mastermix (Life Technologies), forward and reverse primers (900 nM), probe (250 nM) in nuclease free water (Ambion)] per well. The PCR reaction was carried out in a StepOnePlus real-time PCR machine (Life Technologies). Samples were initially heated to 95°C for 20 s followed by 40 cycles of heating to 95°C for 1 s and cooling to 60°C for 20 s. The efficiency of each primer and probe set was used to calculate the relative mRNA expression ratio using the Pfaffl method (Pfaffl, 2001), normalizing the PCR readouts for the target genes to those of the reference genes.

**Table 1:** **Primers and probe sequences used in RNA analysis, including gene symbol and accession number for the target genes**.

**Gene symbol**	**Accession number**	**Forward primer sequence**	**Reverse primer sequence**	**Probe sequence**
*ActB*	NM_031144.3	GACCCAGATCATGTTTGAGACCTT	AGAGGCATACAGGGACAACACA	AACACCCCAGCCATGTACGTAGCCATC
*Ywhaz*	NM_013011.3	TGCTGCTGGTGATGACAAGAA	CATCTCCTTTTTGCTGATTTCAAA	TGGACCAGTCACAGCAAGCATACCAAGAA
*Hprt1*	NM_012583.2	CCTCCTCAGACCGCTTTTCC	CATAACCTGGTTCATCATCACTAATCA	CATGTCGACCCTCAGTCCCAGCG
*c-Fos*	NM_022197.2	CAGCCAAGTGCCGGAATC	GCAACGCAGACTTCTCGTCTT	ATACGCTCCAAGCGGAGACAGATCAACTT
*Arc*	NM_019361.1	GGGTGGCTCTGAAGAATATTGG	CACCGAGCCCTGTTTGAACT	AGATCCAGAACCACATGAATGGGCCA
*Egr1*	NM_012551.2	AAGACACCCCCCCATGAAC	CTCATCCGAGCGAGAAAAGC	CCCGTATGCTTGCCCTGTTGAGTCC

### Chromatin immuno-precipitation (ChIP)

Rats were killed under baseline conditions or at 30 min after the start of MWM or SC procedures. Due to the limited size of the rat hippocampus and the requirement of sufficient amounts of chromatin, each sample for ChIP was prepared from hippocampus tissue of two rats. Therefore, for the preparation of each chromatin sample, the hippocampus tissue of two rats was cross-linked in 1% formaldehyde solution containing 5 mM sodium butyrate (NaBut), phosphatase inhibitors [1 PhosSTOP Phosphatase Inhibitor Cocktail Tablet/10 ml (Roche)] and 0.1 mM PMSF in 1 x Phosphate-buffered saline (PBS) at RT for 10 min after which glycine was added to a final concentration of 200 mM. The mixture was rotated at RT for 5 min and then centrifuged (5 min, 6000 g, 4°C). The pellet was washed three times (with a centrifuge step between each wash) in ice-cold PBS with 5 mM NaBut, 1 mM Na_3_VO_4_, 5 ng/ml aprotinin, 2 mM AEBSF and phosphatase inhibitors (1 PhosSTOP Phosphatase Inhibitor Cocktail Tablet/10 ml). The pellet was then re-suspended in ice-cold lysis buffer (50 mM Tris-HCl pH 8.0, 150 mM NaCl, 5 mM EDTA pH 8.0, 0.5% v/v Igepal, 0.5% Sodium deoxycholate, 1% SDS, 5 mM NaBut, 2 mM AEBSF, 1 mM Na_3_VO_4,_ 1x dilution of protease inhibitor solution from Complete Ultra EDTA-free protease inhibitor tablets [Roche) and phosphatase inhibitors (1 PhosSTOP Phosphatase Inhibitor Cocktail Tablet/10 ml)] and rotated at 4°C for 15 min.

The lysate was sheared by sonication on high power for three sets of 10 cycles (30 s on and 60 s off) using a cooled Bioruptor (UCD-300; Diagenode). Samples were then centrifuged (10 min, 21,000 g, 4°C) and the supernatant (containing the sheared chromatin) was collected. An aliquot of chromatin was taken from each sample to be prepared and used as input DNA. Other aliquots of chromatin from each sample were 10-fold diluted in dilution buffer (50 mM Tris-HCl pH 7.4, 150 mM NaCl, 5 mM EDTA pH 7.4, 1% triton X-100, 0.1% w/v sodium deoxycholate, 5 mM NaBut, 1 mM AEBSF, 1x dilution of protease inhibitor solution from Complete Ultra EDTA-free protease inhibitor tablets (Roche), phosphatase inhibitors (1 PhosSTOP Phosphatase Inhibitor Cocktail Tablet/10 ml) and then rotated with rabbit polyclonal histone H3 (acetyl K9, phospho S10) antibody (Abcam) overnight at 4°C. This antibody specifically recognizes the combinatorial mark H3K9ac-S10p and presents a higher binding to c-Fos and Egr-1 gene promoters in ChIP assays than the anti-H3S10p antibody (Reul, unpublished observations). These observations in the hippocampus indicate that if H3S10 is phosphorylated, then the neighboring K9 (and K14 Chandramohan et al., 2007) is acetylated, which corresponds with past *in vitro* studies (Barratt et al., 1994). The anti-H3K9ac-S10p antibody, rather than the anti-H3S10p-K14ac antibody, was used for ChIP because recent lots of the latter antibody have lost their stringent specificity for binding the dual H3S10p-K14ac mark (Reul lab, unpublished observations). For each sample, a 100 μl aliquot of PureProteome Protein A magnetic beads (Merck Millipore) was washed twice with 0.5% BSA in 1 x PBS and rotated with 0.5% BSA/PBS overnight at 4°C.

The following day, the antibody-chromatin mixtures were added to the beads and rotated at 4°C for 3 h. Next, the beads were washed three times with ice-cold RIPA buffer (10 mM Tris-HCl pH 7.4, 1 mM EDTA, 0.1% SDS, 0.5 mM EGTA, 1% triton X-100, 0.1% sodium deoxycholate, 140 mM NaCl, 1x dilution of protease inhibitor solution from Complete Ultra EDTA-free protease inhibitor tablets (Roche), phosphatase inhibitors (1 PhosSTOP Phosphatase Inhibitor Cocktail Tablets/25 ml), 1 mM AEBSF, 5 mM NaBut) and twice with ice-cold 1 x Tris-EDTA buffer. The bound fraction was then eluted from the beads in two 15-min elution steps with regular vortexing, using elution buffer 1 (10 mM Tris-HCl pH 7.4, 50 mM NaCl, and 1.5% SDS) and elution buffer 2 (10 mM Tris-HCl pH 7.4, 50 mM NaCl, and 0.5% SDS). The protein-DNA crosslinks were reversed by adding NaCl to a final concentration of 200 mM to the bound samples and the input samples, and incubating at 65°C overnight.

Next, the bound and input samples were incubated sequentially with ribonuclease A from bovine pancreas, (final concentration of 125 μg/ml) for 1 h at 37°C and proteinase K from *Tritirachium album* (final concentration of 125 μg/ml) for 3 h at 37°C. A QIAquick PCR purification kit (Qiagen) was used to purify the immuno-precipitated DNA, as per the manufacturer's instructions. Following this, the concentration of double stranded DNA was measured with a high-sensitivity double-stranded DNA assay kit using a Qubit 2.0 Fluorometer (Life Technologies). Bound DNA product and inputs were diluted to 0.05 ng/μl and 0.5 ng/μl respectively in nuclease free water (nfH_2_O).

Quantitative PCR (qPCR) was used to quantify the levels of target genes within the bound and input fractions using primers and dual-labeled probes modified with FAM as the fluorescent dye and TAMRA as the quencher. Primer and probe sets [designed using primer express software (version 3.0)] used after ChIP are shown in Table 2. Primers and probes were designed to target CREB responsive elements (CREs) in the proximal promoters of the IEGs. qPCR was principally performed as described above; samples (0.1 ng Bound fraction; 1 ng Input fraction) were run in triplicate and a standard curve was performed using serial dilutions of rat brain genomic DNA (100 ng/μl; BioChain, CA, USA) as standards. The amount of target DNA for each sample was calculated by comparing the CT values from the bound and input fractions with the standard curve. After correcting for differential loading (1 ng input compared with 0.1 ng bound), enrichment was calculated using the following equation: Bound fraction/ Input fraction (B/I).

**Table 2:** **Primers and probe sequences used in ChIP analysis, including gene symbol and Ensemble gene ID for the target genes**.

	**Ensemble gene ID**	**Forward primer sequence**	**Reverse primer sequence**	**Probe sequence**
*c-Fos*	ENSRNOG00000008015	TTCCCCCCTCCAGTTTCTCT	TCAGCTGGCCGCTTTATAGAAG	TTCCGCTCATGACGTAG
*Egr1*	ENSRNOG00000019422	GCTCTTGGATGGGAGGTCTTC	TCCGCCGTGACGTACATG	TCCTCCCGGTCGGTCCT
*Arc*	ENSRNOG00000043465	TGAGGGCAAATAGCATGTAATAACC	CAGCCCGGAGTGACTAATGTG	CTTAGCTTCACCCTCGCTGCTCAGGAC

### Statistical analysis

The statistical and graphical package used to analyze data was GraphPad Prism 5 (GraphPad Software, San Diego, CA, USA). The MWM behavioral data were expressed as the average number of seconds taken to find the platform per trial or per day. Differences between trials or days were analyzed using repeated measures One-Way analysis of variance (ANOVA) followed by Dunnett's *post-hoc* test. Performances of SC and MWM groups in the probe trial were compared using unpaired Student's *t*-tests.

Immunohistochemical data were expressed as the average number of immuno-positive neurons counted in a dentate gyrus, or a selected region of CA1 or CA3, in a 50 μm section of the dorsal hippocampus. Three sections were counted, per rat, by an experimenter blind to the treatment of the animal being counted. In the case of Egr-1 staining in the CA1, the majority of neurons appeared immuno-positive for Egr-1, although staining intensity varied between experimental groups. ImageJ software (Schneider et al., 2012) was, therefore, used to analyze the Egr-1 staining in this brain region. The CA1 region was outlined and an average gray value was determined. This gray value was normalized (in order to adjust for any differential levels of development) based on the gray value determined in an un-stained region, representing background staining, adjacent to the pyramidal cell layer.

Relative RNA expression is presented as a fold-change over baseline levels after normalization to reference genes, whilst ChIP data is expressed as fold change in enrichment over baseline levels. Group means ± SEM are shown in the figures and samples sizes are stated in each case in the figure legends. Immunohistochemistry, RNA and ChIP data were statistically analyzed using One-Way ANOVA followed by Dunnett's or Bonferroni *post-hoc* tests where appropriate. Linear regression analysis was used to analyze correlations between numbers of immuno-stained neurons and behavior during MWM training. In all cases, *P* < 0.05 was accepted as statistically significant.

## Results

### Acquisition and retention of the MWM task

**Figure 1:**
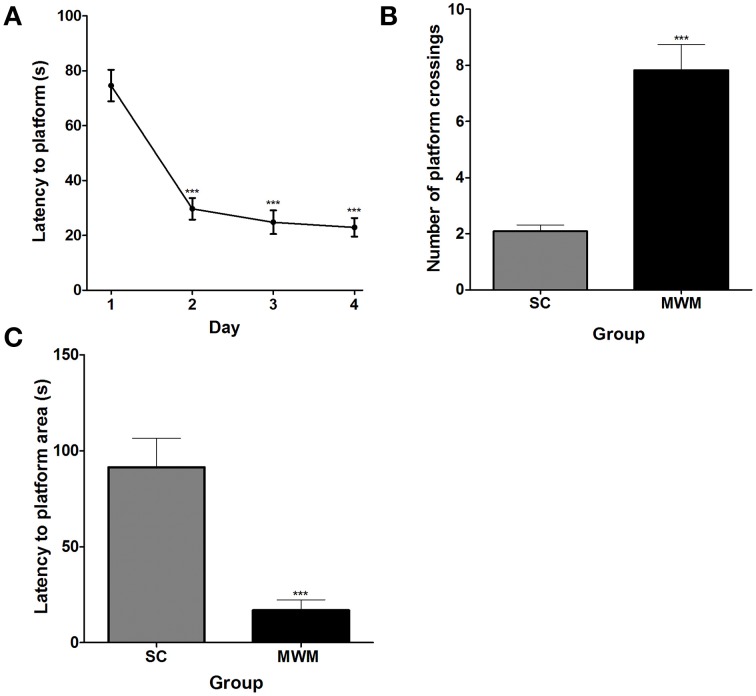
**Performance of rats in the MWM during training and in a probe trial 24 h later. (A)** Average latency (± SEM, *n* = 12) to the platform on each of the four MWM training days (one training session per day, each comprising four training trials). **(B)** Average number of platform zone crossings (± SEM, *n* = 12) recorded for MWM-trained rats during a 3 min long probe trial, 24 h after the final training session, compared with numbers of platform zone crossings in time matched swim control rats which were not trained to find a platform. **(C)** The average latency (± SEM, *n* = 12) to the first crossing of the platform zone during the 3 min probe trial for MWM-trained and SC rats. Statistics: **(A)** Repeated measures ANOVA, *F*_(3, 33)_ = 36.70, *p* < 0.0001, Dunnett's *post-hoc* test ^***^p < 0.001. **(B)** Unpaired Student's *t*-test, *t* = 6.17, *p* < 0.0001. **(C)** Unpaired Student's *t*-test, *t* = 4.59, *p* = 0.0001.

Over the course of 4 days of MWM training, rats learnt the location of the hidden platform, showing decreasing average latencies to the platform across days. The average latency to find the hidden platform was significantly lower on the second, third and fourth day of training than on the first and by the final training day rats were able to find the platform in an average time of 22.9 ± 3.4 s (*n* = 12), compared with an average of 74.6 ± 5.8 s on the first day, indicating that the performance of the rats improved with training (Figure 1A). During the 3-min probe trial in the absence of the platform, MWM-trained rats crossed the platform zone an average of 7.8 ± 0.9 times compared with only 2.1 ± 0.2 times in the SC group (Figure 1B). In addition, the average latency to the first crossing of the platform zone was 16.9 ± 5.4 s in the MWM-trained group, compared with 91.3 ± 15.3 s in the SC group (Figure 1C). As the largest improvement was seen between the first and second training days, all subsequent investigations into the molecular changes occurring in hippocampal neurons during training focused on the first day of training.

### MWM training leads to an increase in the phosphorylation of ERK1/2 and H3S10, and induction of immediate early genes in the dentate gyrus and CA regions

**Figure 2:**
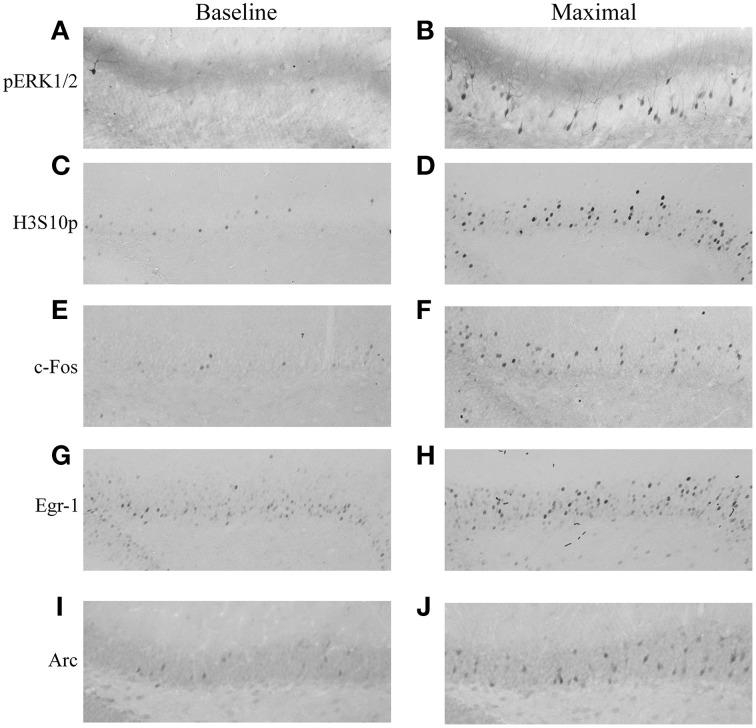
**Immunohistochemical staining of pERK1/2, H3S10p, c-Fos, Egr-1, and Arc in the DG under baseline conditions and after MWM training**. Rats were killed either under baseline conditions or at different stages of MWM training. Representative images of the dorsal blade of the DG are shown for **(A)** pERK1/2 under baseline conditions, **(B)** pERK1/2 at maximal induction (immediately following the acclimatization trial), **(C)** H3S10p under baseline conditions, **(D)** H3S10p at maximal induction (immediately following the second training trial), **(E)** c-Fos under baseline conditions, **(F)** c-Fos at maximal induction (immediately following final training trial), **(G)** Egr-1 under baseline conditions, **(H)** Egr-1 at maximal induction (2 h after the start of training), **(I)** Arc under baseline conditions, **(J)** Arc at maximal induction (2 h after the start of training). Magnification: 200-fold.

The phosphorylation of ERK1/2 and H3S10, and the induction of c-Fos, Egr-1, and Arc was analyzed in the DG, CA1, and CA3 during 1 day of MWM training and up to 8 h later. Immunohistochemical analysis in the dorsal hippocampus showed nuclear and cytoplasmic pERK1/2 staining in granule neurons within the granular cell layer of the DG, with a clear increase in the proportion of immuno-stained neurons after MWM exposure, predominantly observed in the dorsal blade (Figures 2A,B). Immuno-staining for H3S10p, c-Fos and Egr-1 each revealed a sparse, nuclear-specific staining of DG granule neurons at baseline, with increased numbers of immuno-stained neurons and increased staining intensity after MWM training, also predominantly in the dorsal blade (Figures 2C–H). Immuno-staining showed Arc to be both nuclear and cytoplasmic, with increases observed in numbers of immuno-positive neurons in the DG after MWM (Figures 2I,J).

**Figure 3:**
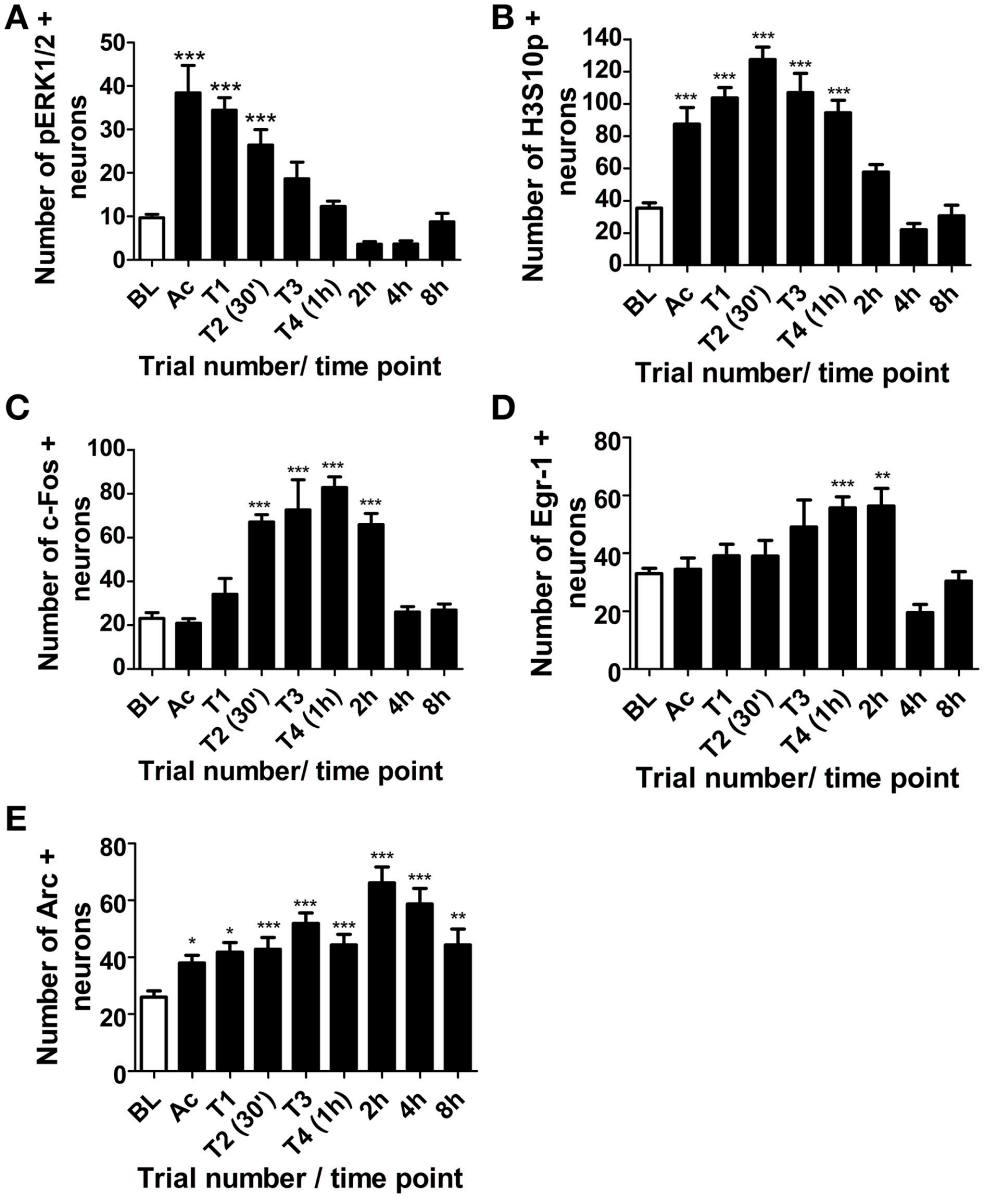
**Changes in pERK1/2, H3S10p, c-Fos, Egr-1 and Arc in the DG in response to MWM training**. Rats were killed either under baseline conditions (BL), immediately following the 3 min acclimatization trial (Ac), immediately following each of the four MWM training trials (T1-T4) or 2, 4, or 8 h after the start of MWM training. The graphs present **(A–E)** the average numbers of neurons (±SEM, *n* = 5–6, except BL and T4 (1 h): *n* = 12) immuno-positive for **(A)** pERK1/2 **(B)** H3S10p, **(C)** c-Fos **(D)** Egr-1, and **(E)** Arc in the DG within a 50 μm thick coronal brain section. Statistical analysis: One-Way ANOVA with Dunnett's *post-hoc* test, **(A)** *F*_(8, 54)_ = 21.99, *p* < 0.0001, **(B)** *F*_(8, 57)_ = 26.07, *p* < 0.0001, **(C)** *F*_(8, 57)_ = 22.66, *p* < 0.0001, **(D)** *F*_(8, 57)_ = 7.59, *p* < 0.0001, **(E)** *F*_(8, 57)_ = 9.28, *p* < 0.0001; Dunnett's *post-hoc* test: ^***^p < 0.001, ^**^p < 0.01, ^*^p < 0.05 significantly different from BL.

Phosphorylation of ERK1/2 in the dentate gyrus was rapid, with a maximal increase in pERK1/2 positive neurons immediately after the initial 3-min acclimatization trial in the MWM. Levels declined steadily throughout training and by the end of the third training trial were no longer significantly different from baseline levels (Figure 3A). Numbers of H3S10p-positive neurons in the DG were also significantly higher than baseline by the end of the initial acclimatization trial, but numbers peaked after the second training trial (approximately 30 min into training) and were still significantly higher than baseline at the end of the final training trial (approximately 1 h after the start of training) (Figure 3B). A significant increase in c-Fos positive neurons in the dentate gyrus was seen between 30 min and 2 h after the start of training, peaking at 1 h, whilst numbers of Egr-1 positive neurons increased significantly above baseline between 1 and 2 h after the start of MWM training (Figures 3C,D). In contrast, numbers of Arc-positive neurons were significantly higher than baseline at all-time points studied, even up to 8 h after the start of training, with peak Arc expression found 2 h after the start of training (Figure 3E).

**Figure 4:**
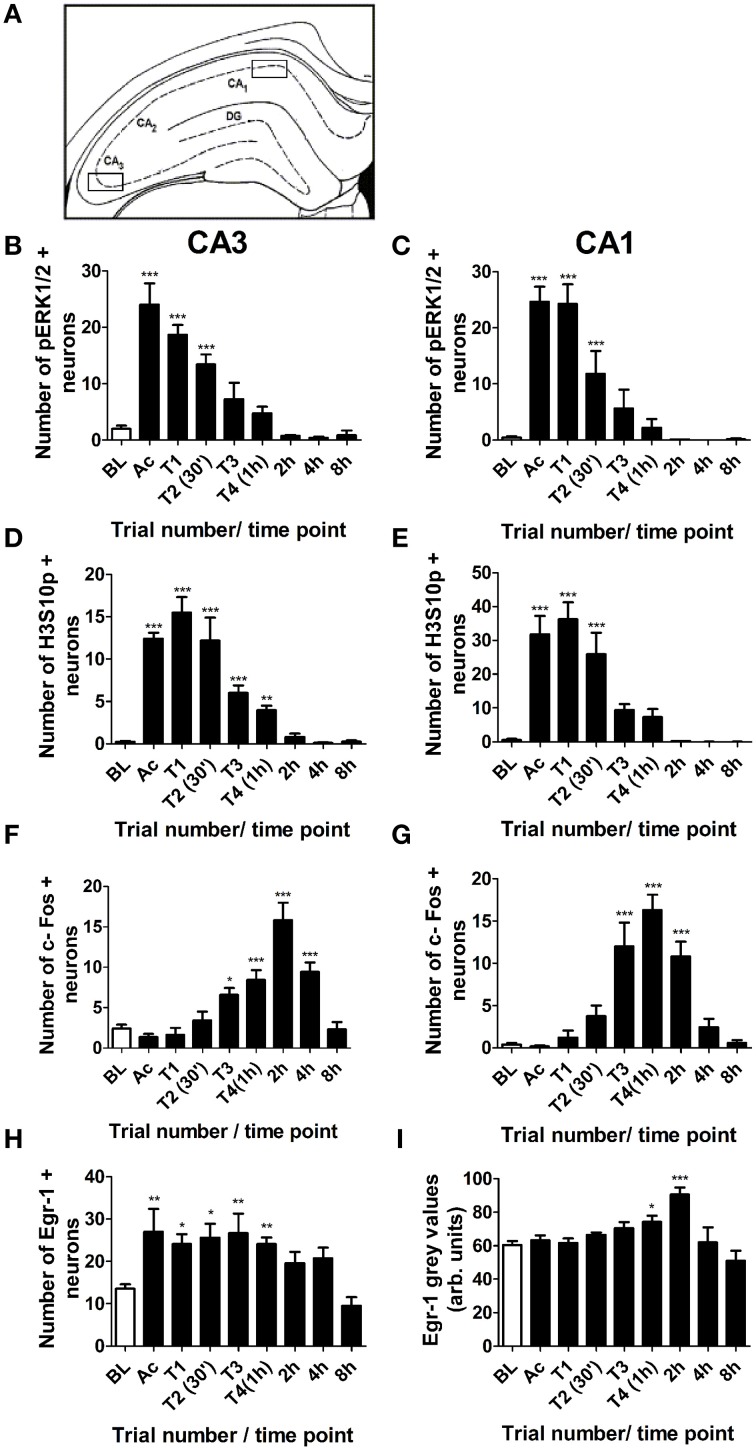
**Changes in pERK1/2, H3S10p, c-Fos and Egr-1 in the CA3 and CA1 in response to MWM training**. Rats were killed either under baseline conditions (BL), immediately following the 3 min acclimatization trial (Ac), immediately following each of the four MWM training trials (T1–T4) or 2, 4, or 8 h after the start of MWM training. The graphs present the average numbers of neurons (±SEM, *n* = 5–6, except BL and T4 (1 h): *n* = 12) in the regions of the CA3 and CA1 shown in **(A)** which were immuno-positive for **(B)** pERK1/2 in the CA3, **(C)** pERK1/2 in the CA1, **(D)** H3S10p in the CA3, **(E)** H3S10p in the CA1 **(F)** c-Fos in the CA3, **(G)** c-Fos in the CA1, **(H)** Egr-1 in the CA3, and **(I)** Egr-1 in the CA1 within a 50 μm thick coronal brain section. Arc staining in the CA regions was indistinct and was therefore not analyzed. Statistical analysis: One-Way ANOVA with Dunnett's *post-hoc* test, **(B)** *F*_(8, 54)_ = 25.02, *p* < 0.0001, **(C)** *F*_(8, 54)_ = 20.08, *p* < 0.0001, **(D)** *F*_(8, 57)_ = 34.83, *p* < 0.0001, **(E)** *F*_(8, 57)_ = 22.06, *p* < 0.0001, **(F)** *F*_(8, 56)_ = 16.63, *p* < 0.0001, **(G)** *F*_(8, 56)_ = 20.87, *p* < 0.0001 **(H)** *F*_(8, 57)_ = 5.10, *p* < 0.0001 **(I)** *F*_(8, 57)_ = 6.10, *p* < 0.0001. Dunnett's *post-hoc* test: ^***^p < 0.001, ^**^p < 0.01, ^*^p < 0.05 significantly different from baseline.

Immunohistochemical staining for pERK1/2, H3S10p, c-Fos, Egr-1 and Arc was also analyzed in the CA3 and CA1 regions of the hippocampus. Numbers of immuno-positive neurons were counted from one microscope view (x 20 magnification) within the CA1 and CA3, as shown in Figure 4A. Changes in numbers of pERK1/2 immuno-positive neurons in the CA3 and CA1 were similar to the DG, with numbers peaking immediately after the acclimatization trial and gradually declining throughout training (Figures 4B,C). Staining for phosphorylated H3S10 in the CA regions was less intense than in the DG and occurred later, peaking after the first training trial and remaining significantly higher than baseline for 1 h in the CA3 region and 30 min in the CA1 region (Figures 4D,E). The numbers of c-Fos-positive neurons increased above baseline levels after the third training trial and peaked at 1 h in the CA1 and at 2 h in the CA3 (Figures 4F,G). In contrast to the DG, Egr-1 showed a very rapid increase in the CA3 and numbers of immuno-positive neurons were significantly higher than baseline for the duration of training (Figure 4H). Egr-1 staining in the CA1 region was visible in most cells at baseline, with an increase in staining intensity between 1 and 2 h after the start of MWM training (Figure 4I). Arc immunostaining in the CA regions was not analyzed as staining was not discernible and did not appear to differ from baseline at any time point investigated.

As we have previously shown that glucocorticoid hormones via GRs facilitate ERK MAPK signaling to the chromatin (Gutierrez-Mecinas et al., 2011), changes in plasma corticosterone levels in the MWM paradigm were investigated. Training in the MWM led to a significant increase in plasma corticosterone levels, which peaked at the end of training (approximately 1 h; Figure S1A). By 4 h, plasma corticosterone levels were no longer significantly higher than baseline, and by 8 h plasma corticosterone levels had risen again over baseline levels, due to normal circadian rhythm (Figure S1A).

### The number of H3S10p–positive neurons in the DG after training positively correlates with the average latency to find the platform during training

**Figure 5:**
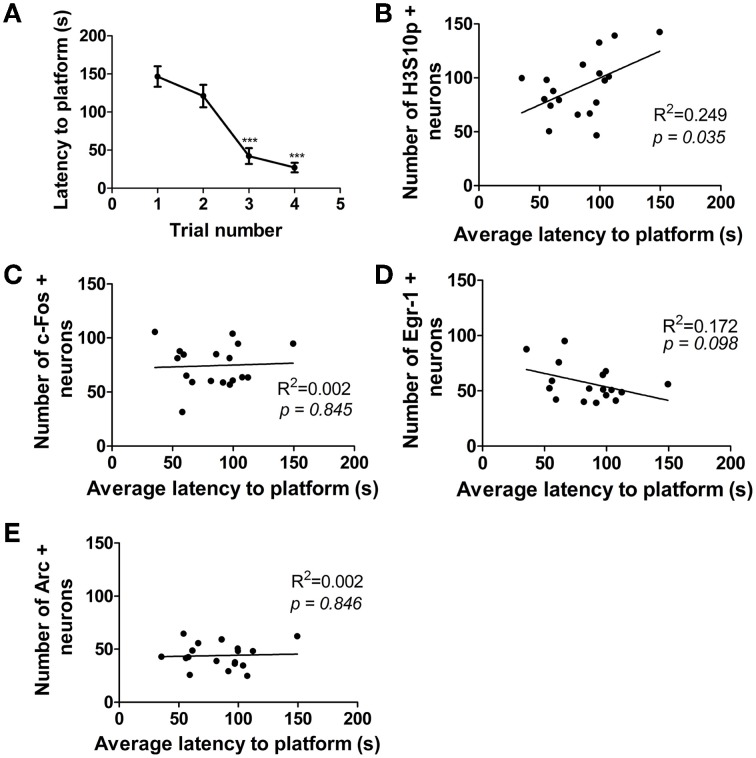
**Correlation between performance in the MWM and numbers of immuno-positive neurons in the DG**. Rats were given one training session (acclimatization trial plus four training trials) and were killed immediately after the final trial (approximately 1 h after the start of training). **(A)** Average latency (± SEM, *n* = 18) to the platform on each of the four MWM training trials. Average latency to platform across all four trials was plotted against numbers of neurons in the DG immuno-positive for **(B)** H3S10p, **(C)** c-Fos, **(D)** Egr-1, and **(E)** Arc. Values plotted are for individual rats (*n* = 17–18). Statistical analysis: **(A)** repeated measures ANOVA: *F*_(3, 51)_ = 27.18, *p* < 0.0001, Dunnett's *post-hoc* test ^***^p < 0.001 **(B–E)** Linear regression: **(B)** *R*^2^ = 0.249, *p* = 0.035, **(C)** *R*^2^ = 0.002, *p* = 0.845, **(D)** *R*^2^ = 0.172, *p* = 0.098, **(E)** *R*^2^ = 0.002, *p* = 0.846.

Over the course of 1 day of MWM training, the time taken for the rats to find the hidden platform decreased significantly (Figure 5A), however, there was some variation in performance between individual rats. A significant, positive correlation was found between average latency to find the platform over the course of 1 day of training and the numbers of neurons immuno-positive for H3S10p at the end of training (1 h time point) in the DG (Figure 5B). There was no such correlation between performance in the MWM and numbers of c-Fos, Egr-1, or Arc immuno-positive neurons (Figures 5C–E). There was also no correlation between MWM performance and numbers of neurons immuno-positive for any of these factors in either the CA3 or the CA1 regions of the hippocampus (data not shown).

### Hippocampal H3S10p and IEG changes in MWM-trained rats are highly similar to the changes occurring in SC rats

**Figure 6:**
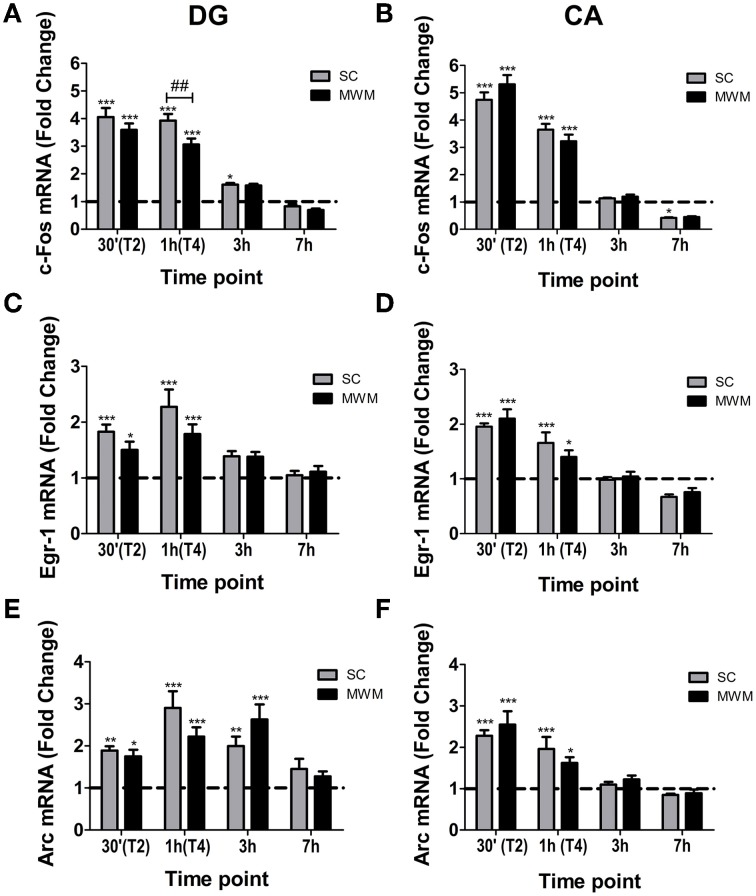
**IEG mRNA changes in the hippocampus in MWM trained or swim control rats following 1 day of training**. Rats were either killed under baseline conditions (BL) or 30 min, 1 h (immediately after final trial), 3 h or 7 h after the start of the first day of MWM training or swim control procedure. The graphs show the mean fold change over BL (±SEM, *n* = 5–9) mRNA levels of **(A)** *c-Fos* in the DG, **(B)** *c-Fos* in the CA regions, **(C)** *Egr-1* in the DG, **(D)** *Egr-1* in the CA regions, **(E)** *Arc* in the DG, **(F)** *Arc* in the CA regions. BL is indicated by a dashed line. Relative mRNA expression was calculated following qPCR with the Pfaffl method. Statistical analysis: One-Way ANOVA; **(A)** *F*_(8, 52)_ = 82.09, *p* < 0.0001, **(B)** *F*_(8, 54)_ = 150.8, *p* < 0.0001, **(C)** *F*_(8, 52)_ = 11.53, *p* < 0.0001, **(D)** *F*_(8, 52)_ = 30.61, *p* < 0.0001, **(E)** *F*_(8, 52)_ = 12.93, *p* < 0.0001, **(F)** *F*_(8, 54)_ = 20.46, *p* < 0.0001. Bonferroni *post-hoc* test: ^***^p < 0.001, ^**^p < 0.01, ^*^p < 0.05 significantly different from BL. ##*p* < 0.01 significantly different from SC at the same time point.

IEG mRNA levels were measured 30 min, 1, 3, and 7 h after the start of MWM training and compared with mRNA levels in matched SC rats. In both the DG and CA regions, *c-Fos* mRNA levels were increased in MWM trained and SC groups (Figures 6A,B). At 1 h after the start of training DG *c-Fos* levels in the SC group were significantly higher than in the MWM group (fold changes over baseline; 3.9 ± 0.2 and 3.1 ± 0.2 respectively). *Egr-1* mRNA levels were significantly increased over baseline levels in the DG and CA regions in both SC and MWM groups at 30 min and 1 h, before returning to baseline at 3 h (Figures 6C,D). *Arc* mRNA was also up-regulated in the DG and CA regions in both SC and MWM groups (Figures 6E,F). In the DG, the up-regulation was extended, with *Arc* mRNA levels still significantly higher than baseline 3 h after training. There were no significant differences in *Egr-1* or *Arc* mRNA levels between SC and MWM groups at any time point studied in either the DG or CA regions.

Immunohistochemical analyses were carried out on dorsal hippocampal slices taken from MWM-trained and SC rats at 30 min and 1 h post training, in order to compare levels of H3S10p and IEG proteins in hippocampal neurons between the two experimental conditions. There were, however, no significant differences between SC and MWM groups in the numbers of immuno-positive neurons at either time point (Table 3).

**Table 3:** **Immuno-positive neurons in hippocampal sub-regions after MWM or SC experiences**.

**Number of immuno-positive neurons**							
		**BL**	**SC 30′**	**MWM 30′**	**SC 1 h**	**MWM 1 h**	**Statistics**
DG	H3S10p	35.2 ± 3.7	86.8 ± 3.8^*^	98.1 ± 6.3^*^	79.7 ± 6.3^*^	87.4 ± 12.6^*^	*F*_(4, 25)_ = 11.24 *P* < 0.0001
	c-Fos	15.4 ± 1.8	37.0 ± 3.0^*^	40.0 ± 3.7^*^	51.7 ± 4.2^*^	57.2 ± 5.2^*^	*F*_(4,25)_ = 19.98 *P* < 0.0001
	Egr-1	39.9 ± 7.5	23.8 ± 6.5	32.2 ± 9.7	42.5 ± 7.1	60.2 ± 10.8	*F*_(4, 24)_ = 2.49 *P* = 0.0705
	Arc	17.5 ± 2.2	45.7 ± 4.5^*^	32.9 ± 3.3^*^	46.9 ± 2.8^*^	43.1 ± 4.3^*^	*F*_(4, 25)_ = 12.11 *P* < 0.0001
CA3	H3S10p	0.4 ± 0.2	9.1 ± 2.2^*^	10.9 ± 2.3^*^	4.9 ± 0.6	4.6 ± 1.2	*F*_(4, 24)_ = 7.68 *P* = 0.0004
	c-Fos	2.6 ± 0.7	3.0 ± 0.7	4.2 ± 1.3	7.1 ± 1.5	9.3 ± 2.5^*^	*F*_(4, 25)_ = 3.80 *P* = 0.0151
	Egr-1	15.9 ± 2.4	28.8 ± 2.9	29.5 ± 3.5	29.9 ± 4.0	33.5 ± 4.8^*^	*F*_(4, 23)_ = 3.15 *P* = 0.7234
CA1	H3S10p	0.3 ± 0.2	17.1 ± 5.9^*^	19.5 ± 3.4^*^	7.4 ± 2.2	5.1 ± 1.6	*F*_(4, 24)_ = 7.29 *P* = 0.0005
	c-Fos	0.3 ± 0.2	4.0 ± 1.3	3.9 ± 2.3	8.9 ± 1.7^*^	10.2 ± 1.9^*^	*F*_(4, 25)_ = 6.03 *P* = 0.0015
	Egr-1	60.9 ± 2.9	66.86 ± 1.1	62.7 ± 2.0	67.2 ± 2.7	74.9 ± 2.3^*^	*F*_(4, 25)_ = 5.48 *P* = 0.0028

Rats were killed at baseline, 30 min or 1 h after the start of either MWM or SC procedures. Immunohistochemistry for H3S10p, c-Fos, Egr-1, and Arc was performed on coronal brain sections. Values with regard to the “Number of immuno-positive neurons” are the mean number of immuno-positive neurons counted from the named region (± SEM, n = 5–6). Statistical analysis: One-Way ANOVA followed by Bonferroni post-hoc test. Data indicated with a

* *signifies a p < 0.05 significant difference from baseline (BL). There were no significant differences between SC and MWM at any time point. “Statistics” indicates the ANOVA result*.

Plasma corticosterone levels were increased significantly above baseline in both MWM and SC groups, but there were no significant differences between MWM and SC groups at either time point studied (Figure S1B).

### There is increased association of H3K9ac-S10p with *c-Fos* and *Egr-1* gene promoters after SC and MWM

**Figure 7:**
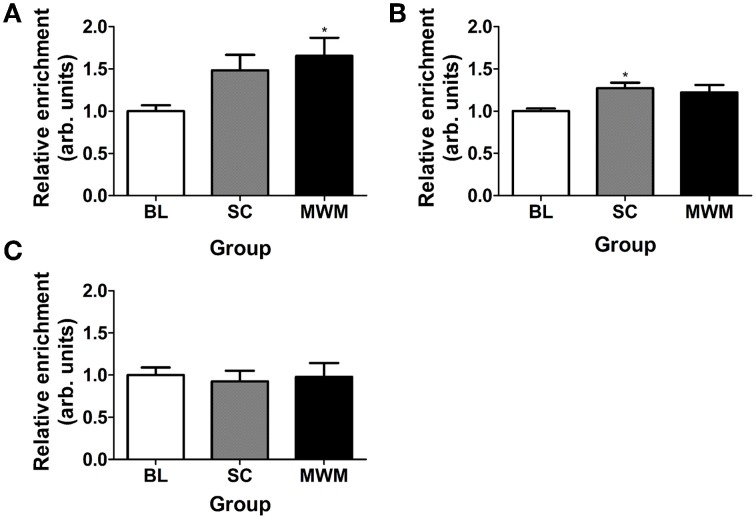
**Changes in enrichment of H3K9ac-S10p at the promoters of *c-Fos, Egr-1*, and *Arc* after SC and MWM**. Rats were killed either under baseline conditions or 30 min after the start of the first day of MWM training or swim control procedure. Chromatin immuno-precipitation was carried out on whole hippocampus tissue. The graphs show the relative enrichment of H3K9ac-S10p at **(A)** the *c-Fos* promoter, **(B)** the *Egr-1* promoter, **(C)** the *Arc* promoter. The enrichment for each group was calculated by dividing the quantity of target DNA in the H3K9ac-S10p-bound fraction by the quantity of DNA in the input fraction (bound over input, B/I) and data is expressed as a ratio over baseline enrichment [±SEM, *n* = 3–6 (every replicate comprised hippocampal chromatin from two rats)]. Statistical analysis: One-Way ANOVA; **(A)** *F*_(2, 15)_ = 3.98, *p* = 0.0369, **(B)** *F*_(2, 14)_ = 4.06, *p* = 0.0407, **(C)** *F*_(2, 15)_ = 0.08, *p* = 0.9204. Dunnett's *post-hoc* test: ^*^p < 0.05 significantly different from baseline (BL).

ChIP for the dual histone mark H3K9ac-S10p was carried out using chromatin of whole hippocampus tissues collected 30 min after the start of MWM or SC. We found a significant increase in the association of H3K9ac-S10p at the promoters of the IEGs *c-Fos* and *Egr-1*, whilst levels of the dual histone mark at the *Arc* promoter remained unchanged across all conditions (Figure 7).

## Discussion

**Figure 8:**
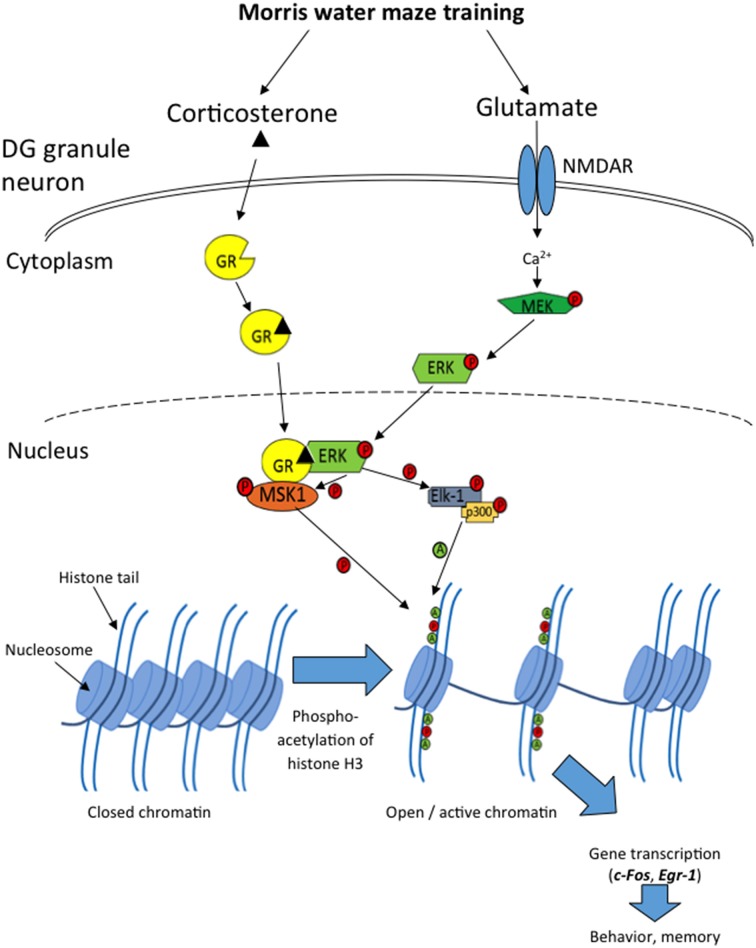
**Diagram showing the cascade of events in sparse DG neurons following MWM training, leading to c-Fos and Egr-1 transcription**. Introducing a rat to the MWM activates NMDARs, causing initiation of the MAPK/ERK cascade within the neuron, leading to phosphorylated ERK1/2 entering the nucleus. Concurrently, glucocorticoid hormone-occupied GR also translocates to the nucleus where it physically interacts with pERK1/2, allowing the phosphorylation of the nuclear kinases MSK1 and Elk-1. MSK1 then phosphorylates histone H3 at serine 10, and Elk-1recruits and phosphorylates p300, a histone acetyl transferase, which acetylates histone H3 at lysine 9, lysine 14 and possibly other lysine residues. These modifications result in the opening of the chromatin structure and facilitation of transcription of genes such as c-Fos and Egr-1, which are involved in memory consolidation and behavioral responses to the MWM.

The present study shows that exposure to the MWM leads to a rapid, transient phosphorylation of pERK1/2, a more protracted phosphorylation of H3S10, and transcription and translation of the IEGs c-Fos, Egr-1, and Arc predominantly in sparse neurons of the DG. The time course of induction of each of these components is in line with the concept of rapid ERK/MAPK activation leading to phospho-acetylation of histone H3, which in turn alters chromatin conformation allowing induction of c-Fos and Egr-1 (Figure 8); a sequence of events also shown to occur in the DG in response to forced swimming (Chandramohan et al., 2008; Gutierrez-Mecinas et al., 2011). We have previously shown that these molecules are co-localized within the same DG granule neurons after forced swimming or novelty exposure (Chandramohan et al., 2007; Gutierrez-Mecinas et al., 2011). Therefore, it is likely that this cascade of events also occurs within specific DG neurons following MWM training.

In addition to the changes seen in the DG, phosphorylation of ERK1/2 and H3S10 was also observed in sparse neurons of the CA3 and CA1 regions (although staining for H3S10p in the CA regions was much fainter than in the DG). The IEGs c-Fos and Egr-1 were also induced in the CA regions, however, there was no visible change in Arc. Importantly, using ChIP, we showed an increase in the association of phospho-acetylated histone H3 with the promoter regions of c-Fos and Egr-1 after MWM training, implying a direct role of this epigenetic modification in activity-dependent transcription of these genes.

The H3S10p epigenetic mark has been found to form solely in the DG in response to FS and novelty stress (Chandramohan et al., 2007, 2008; Gutierrez-Mecinas et al., 2011); however, the current study has shown that this mark is also increased in CA3 and CA1 pyramidal neurons after MWM training, albeit to a lower staining intensity in each neuron. Our observations correspond with earlier findings of David Sweatt and colleagues who, using western blot analysis, observed H3S10p formation in the mouse CA1 region after contextual fear conditioning (Chwang et al., 2006). It may be speculated that MWM training, due to its repetitive nature, is a stronger stimulus for phospho-acetylation of H3S10 than FS. This notion is supported by a study by Sassone-Corsi and colleagues, who showed an increase in H3S10 phosphorylation in the CA1 and CA3, in addition to the DG, after kainate injection (Crosio et al., 2003); in further agreement with our study, they found staining for H3S10p in the CA regions to be much less intense than in the DG. Notably, our study shows how rapidly H3S10 can be phosphorylated, with a significant increase in H3S10p positive neurons seen even after the initial 3-min acclimatization trial.

The spatio-temporal pattern of c-Fos activation followed a very similar pattern in the DG, CA1 and CA3, however, this was not the case for Egr-1 and Arc. Expression of Egr-1 was distinct in each hippocampal sub-region. In the DG, Egr-1 showed a characteristic sparse nuclear staining, with an increase in the number of stained neurons seen 1–2 h after the start of training, whereas in the CA1 region, constitutive levels of Egr-1 were high, with staining visible in most cells, the intensity of which increased 1–2 h after the start of training. In the CA3 region, baseline levels of Egr-1 appeared similar to those in the DG, however, a very rapid (<5 min) increase in Egr-1 positive neurons was seen, which lasted for the duration of training. It is possible that this finding may reflect roles of Egr-1 in the different functions of the hippocampal sub-regions. For example, as CA3 neurons form an attractor network, involved in generating very rapid representations of novel information (Kesner, 2007; Rolls, 2007), it is possible that the rapid increase in Egr-1 in CA3 neurons may play an important role in this process. The underlying mechanism behind such a rapid increase in Egr-1 protein is not known, however, a rapid increase in Egr-1 protein can be MAPK independent (Revest et al., 2005), and may represent a readily available pool of Egr-1 mRNA which can be rapidly translated in response to experience.

Although *Arc* mRNA increases were seen in the CA regions, Arc protein immuno-staining in the CA regions was indistinct and no visible change could be seen after MWM training. Arc mRNA in the CA regions is transported into the dendrites to be translated locally (Link et al., 1995; Steward et al., 1998; Steward and Worley, 2001), however, other studies have identified nuclear Arc protein staining in pyramidal neurons after exploration of a novel environment (Ramirez-Amaya et al., 2005; Vazdarjanova et al., 2006). The reason for this discrepancy is not known. In the DG, increases in numbers of Arc-positive neurons remained significantly higher than baseline even 8 h after MWM training. Such an extended Arc activation agrees with other studies which also found Arc in the DG to remain high 8 h after spatial exploration (Ramirez-Amaya et al., 2005; Vazdarjanova et al., 2006). Post 8 h time points would be necessary to ascertain just how long Arc remains elevated above baseline in DG neurons after MWM training. In addition to the role of Arc in cytoskeletal organization in the synapse, evidence is accumulating for a nuclear site of action of this IEG, namely that it may be involved in transcriptional regulation through interaction with the histone acetyl-transferase Tip60 (Wee et al., 2014).

The number of H3S10p- positive neurons in the DG 1 h after the start of training showed a significant positive correlation with the average latency to the platform during training. H3S10p is known to respond to psychological stressors including predator stress, novelty and forced swim stress, and not to physical stressors such as ether and cold exposure (Bilang-Bleuel et al., 2005; Chandramohan et al., 2007, 2008). It is therefore unlikely that the increased H3S10p in rats which took longer to find the platform is a result of the additional physical exertion. Previous observations have indicated that the magnitude of a DG H3S10p response is dependent on the intensity of the stimulus (higher light intensity during novel environment exposure and lower water temperature during forced swimming leading to H3S10p-K14ac formation in a larger proportion of neurons) (Bilang-Bleuel et al., 2005; Chandramohan et al., 2007, 2008). Furthermore, pre-treatment of rats with the anxiogenic drug FG7142 enhances the H3S10p-K14ac response to novelty in DG neurons (Papadopoulos et al., 2011). It is possible that a longer time in the maze, and therefore a more protracted exposure to an aversive condition (causing more stress, anxiety, and/or frustration), may prove to be a greater stimulus for neurons to undergo this specific histone modification. Whether this is the result of enhanced ERK MAPK signaling to the chromatin and/or increased glucocorticoid hormone secretion, or an as yet unknown mechanism would, however, need further investigation.

Unlike H3S10p, there was no correlation between performance in the maze and number of neurons expressing c-Fos, Egr-1, or Arc. This observation does not correspond with a previous study showing a significant negative correlation between latency to platform and Arc mRNA levels in the dorsal hippocampus (Guzowski et al., 2001). The studies are not entirely comparable, however, as RNA was measured in the dorsal hippocampus in the Guzowski et al. study, whereas we analyzed Arc protein expression specifically in DG neurons. Considering the significant correlation between H3S10p and the time spent in the maze, the lack of significant correlation between c-Fos and Egr-1 expression and behavioral performance was puzzling. It is particularly surprising given the high similarity in H3S10p, c-Fos, and Egr-1 responses in DG neurons after MWM (present study), FS and novelty stress (Chandramohan et al., 2007, 2008). Moreover, our ChIP experiments have shown the association of H3K9ac-S10p with the c-Fos and Egr-1 gene promoters. Thus, it seems that despite the similarity in response pattern, additional factors (e.g., protein stability, control of mRNA translation) are apparently playing an important role in shaping the IEG response in relation to behavioral performance.

There was remarkably little difference in IEG responses (either mRNA or protein) between MWM rats and SC rats, with the single exception of c-Fos, which was transiently higher in the SC group than the MWM group 1 h after the start of MWM training. Furthermore, there were no significant differences in the numbers of H3S10p positive neurons or in plasma corticosterone responses between the SC and MWM groups. Thus, phospho-acetylation of histone H3 and subsequent IEG activation is not selectively altered when a rat is learning the position of a target location within an environment. Instead, IEG expression was induced comparably in rats simply left to explore the environment, with no specific goal or task. This finding is in agreement with other studies which found no selective MWM changes in IEGs in the dorsal hippocampus compared with swim controls, procedural controls or visible platform controls (Guzowski et al., 2001; Shires and Aggleton, 2008). In contrast, Feldman et al. (2010) observed differential responses in IEG expression between MWM and swim controls, however, their swim controls swam only once which cannot be regarded as matched to the MWM condition (Feldman et al., 2010). In our study, the swim controls were exactly matched to the MWM animals in terms of both number and duration of swims.

Given the many studies that applied antisense oligodeoxynucleotide (ODN) technology and gene deletion models and found that IEGs play a critical role in spatial learning (Paylor et al., 1994; Guzowski et al., 2000; Jones et al., 2001; Guzowski, 2002; Plath et al., 2006; Czerniawski et al., 2011), it is surprising that MWM learning does not involve a greater induction of these genes in the hippocampus than the less complex swim control experience. Apparently, learning (e.g., the layout of the MWM room, the size and shape of the maze, the position of the cues) and the experience of stress is taking place in both groups, leading to associated IEG induction. Notably, learning can only be experimentally tested in the MWM groups but this doesn't exclude that the swim controls learn from their experience as well. In fact, swim control rats change their behavior across repeated trials in the maze (e.g., reduced speed and reduced thigmotaxis); this behavior implies reduced anxiety and indicates that they are learning from previous trials that escape around the walls of the maze is not possible (Carter et al., unpublished observation).

The dual histone mark H3K9ac-S10p showed enrichment at the gene promoter regions of c-Fos and Egr-1, which significantly increased after SC or MWM exposure. This finding strongly indicates that this dual histone mark may be specifically involved in the induction of IEG transcription in response to introduction to the maze. Previously, we showed the association of the H3S10p mark with the c-Fos promoter in the forced swim model (Gutierrez-Mecinas et al., 2011). This association was specifically found in the hippocampus and not found in the neocortex, despite the fact that the neocortex also strongly expresses c-Fos after stress. Furthermore, although there was enrichment of H3K9ac-S10p at the Arc gene promoter in all three experimental groups, there was no significant change after SC or MWM. This suggests that either phospho-acetylated histone H3 is not involved in the induction of this IEG or that the phospho-acetylated state at baseline is sufficient to allow gene induction if challenged.

Previously, we have postulated that whilst H3 phospho-acetylation allows the opening of the chromatin structure, binding of activated transcription factors like phosphorylated CREB to their recognition binding sites within the gene promoter is required for actual gene induction (Mifsud et al., 2011; Trollope et al., 2012). The H3K9ac-S10p ChIP results for c-Fos, Egr-1, and Arc imply that the role of H3K9ac-S10p in gene regulation may be highly gene specific. Further insight into differential association of H3K9ac-S10p with genes associated with differential gene expression in the MWM model may be expected from ChIP combined with next-generation sequencing and RNA sequencing. From the present study we can conclude that, similar to the forced swim model, exploration of the MWM involves activation of the ERK MAPK pathway, formation of H3K9ac-S10p and induction of the IEGs c-Fos and Egr-1 (Gutierrez-Mecinas et al., 2011; Reul, 2014; Reul et al., 2015).

To sum up, phosphorylation of S10 and acetylation of K9 and K14 in histone H3, and induction of the IEGs c-Fos, Egr-1, and Arc occurs in sparse DG granule in response to exploration of the maze, regardless of whether or not the rat is actively learning a platform location. The phospho-acetylation of histone H3 is attuned to the stimulus intensity (in this case the length of time spent in the maze) and is directly associated with the c-Fos and Egr-1 promoters after exploration in the maze. There was very little difference either in IEG expression or in the extent of association of H3K9ac-S10p with the IEG promoters between the MWM trained and swim control groups. This remains a conundrum in the face of the well-known critical dependence of behavioral performance in the MWM model on these IEGs from ODN and gene deletion studies. Therefore, we postulate that distinct AP1- and Egr-1-induced (second wave) genes may shape specific spatial learning-associated molecular processes in hippocampal neurons.

### Conflict of interest statement

The authors declare that the research was conducted in the absence of any commercial or financial relationships that could be construed as a potential conflict of interest.
