# Supplementary material for: Distinct epigenetic and gene expression changes in rat hippocampal neurons after Morris water maze training
Source: Front Behav Neurosci. 2015 Jun 16;9:156. doi: 10.3389/fnbeh.2015.00156 (PMC4468857; doi:10.3389/fnbeh.2015.00156)
Supplement: Supplementary file 1 [file DataSheet1.PDF]

Supplementary Material

Distinct epigenetic and gene expression changes in rat hippocampal neurons after Morris water maze training

S.D. Carter, K.R. Mifsud, J.M.H.M. Reul*

Neuro-Epigenetics Research Group, School of Clinical Sciences, University of Bristol, Bristol, UK

***Correspondence:** Professor Johannes M.H.M. Reul, Neuro-Epigenetics Research Group, School of Clinical Sciences, University of Bristol, Whitson Street, Bristol, BS1 3NY, UK.
hans.reul@bristol.ac.uk.

Supplementary materials and methods

Radioimmunoassay

Plasma corticosterone levels were determined using radio-immuno assays (RIAs) carried out on plasma prepared from blood samples collected at the time of death. An ImmuChem™ Double Antibody Corticosterone ^{125}I RIA kit for rats and mice (MP Biomedicals, New York, USA) was used and the RIA was carried out as per manufacturer's instructions. The detection limit was 2.5 ng/ml and the inter- and intra-assay variations were 6.3 % and 5.7 % respectively.

Supplementary Figure

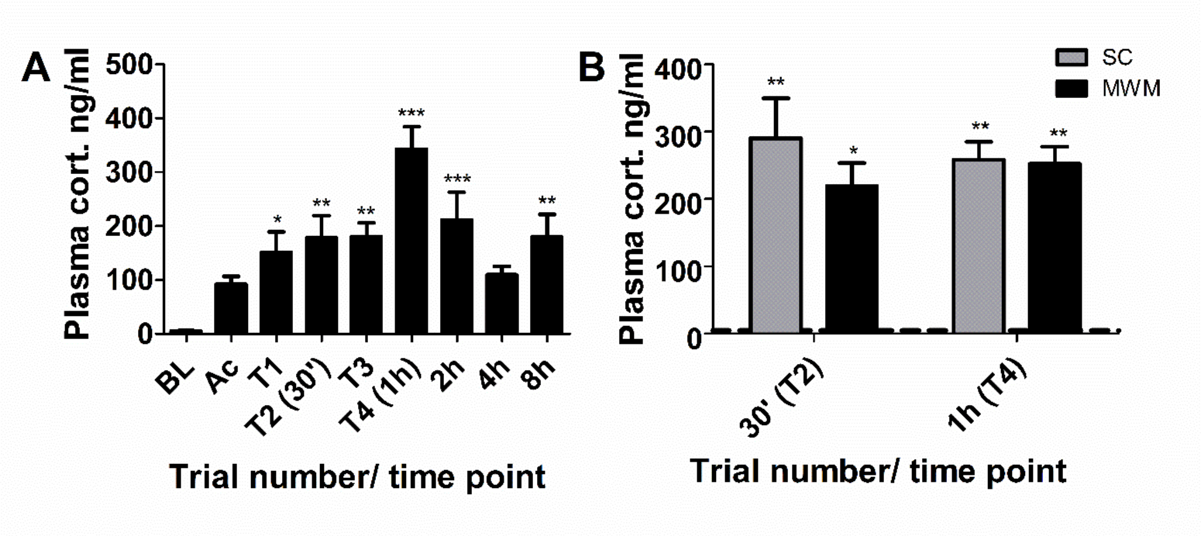

Supplementary Figure 1. Changes in plasma corticosterone levels in response to MWM training and SC procedure. Blood samples were taken, at the time of death, from rats under baseline conditions (BL), immediately following the three minute acclimatization trial (Ac), immediately following each of the four MWM training trials (T1-T4) or 2 hours, 4 hours or 8 hours after the start of MWM training. Further samples were also taken from rats 30 minutes or 1 hour after the start of either MWM or SC procedures. The graphs present the average plasma corticosterone levels in (A) rats trained in the MWM (±SEM, n=5-6, except BL and T4 (1h): n=12), (B) rats trained in the MWM in comparison to swim controls (±SEM, n=6). Statistical analysis: One-way ANOVA (A) $F_{(8,56)}=11.21$, $p<0.0001$, Dunnett's *post-hoc* test, (B) $F_{(4,25)}=10.98$, $p<0.0001$, Bonferroni *post-hoc* test. *** $p<0.001$, ** $p<0.01$, * $p<0.05$ significantly different from BL.
